# Influences on Transmasculine People’s Experiences of Contraceptive Methods and Services in the UK: One-on-One Interviews and a Focus Group

**DOI:** 10.3390/healthcare14152388

**Published:** 2026-08-04

**Authors:** Jessica Madden, Siân Beddows, Richard Ma, Rebecca L. Mawson

**Affiliations:** 1 Primary Care Research, Division of Population Health, School of Medicine and Population Health, University of Sheffield, Regent Court, Sheffield S1 4DA, UK; 2 Centre for Academic Primary Care, Bristol Medical School, University of Bristol, Room 1.07, Canynge Hall, 39 Whatley Road, Bristol BS8 2PS, UK; richard.ma@bristol.ac.uk

**Keywords:** transmasculine, contraception, reproductive healthcare

## Abstract

**Highlights:**

**What are the main findings?**

- This is one of the first UK-based qualitative studies of transmasculine people’s contraceptive experiences, revealing that contraceptive methods are deeply intertwined with gender identity and dysphoria, and cannot be separated from processes of gender affirmation and embodied self-understanding.
- Transmasculine people face significant barriers within NHS contraceptive services. These include misgendering, lack of provider knowledge, and structurally gendered systems, leading many to rely on peer networks rather than healthcare professionals for contraceptive guidance.

**What are the implications of the main findings?**

- Clinicians must move beyond binary assumptions and adopt person-centred approaches that recognise contraceptive decision-making as shaped by gender identity, dysphoria, and lived experience, not solely by clinical need.
- NHS policymakers and guideline developers should address structural barriers which include binary-gendered medical records and the absence of trans-affirming reproductive health guidance.

**Abstract:**

**Background:** Transmasculine people face distinct challenges in accessing and using contraceptive methods, influenced by gendered embodiment, healthcare systems, and identity-driven decision-making. UK-based qualitative research on this topic is minimal. This study aims to gain a deeper insight into the factors that influence transmasculine people’s experiences of contraceptive methods and services in the UK. **Methods:** A qualitative study of semi-structured one-to-one interviews (n = 6) and a focus group (n = 4) was conducted with transmasculine people aged 18+ in the UK, using reflexive thematic analysis informed by Braun and Clarke’s approach. The sample was small and demographically narrow (all participants were white British and based in one UK region), which is considered when interpreting the findings below. **Findings:** Three overarching themes were identified: (1) Gender Identity, Dysphoria, and Reproductive Embodiment; (2) Experiences of Healthcare Systems; and (3) Contraceptive Decision-Making and Use. Participants described profound interactions between dysphoria, bodily autonomy, and contraceptive choice, alongside widespread barriers within healthcare including misgendering, lack of knowledge, and systemic gatekeeping. **Conclusions:** Contraceptive methods are not neutral, they are deeply intertwined with experiences of gender, identity, and power. Findings underscore the urgent need for non-assumptive, person-centred care and contribute new UK-based evidence to inform more equitable reproductive healthcare.

## 1. Introduction

Recent political and social discourse has focussed attention on, and visibility of Lesbian, Gay, Bisexual, Transgender, Queer, and others (LGBTQ+) communities, including transmasculine people. The 2021 UK Census was the first to include a question on gender identity, reporting that in England and Wales, 48,000 people (0.10%) identified as a transgender man and 30,000 (0.06%) as non-binary [1]. Over the last century, gender-affirming care has evolved through the pioneering work of physicians like Hirschfeld and Benjamin, leading to the establishment of organisations, such as the World Professional Association for Transgender Health, that continue to shape modern standards. Despite recent political and social pressures threatening this progress, ensuring equitable access to care for transgender and gender-diverse people remains vital to fostering a more inclusive society [2]. There are still significant gaps which include the provision for contraceptive care for transmasculine people [3].

Transmasculine people (those assigned female at birth who identify on the masculine spectrum, whether as transgender men, non-binary, genderqueer, or other identities) may use contraceptive methods for multiple purposes beyond pregnancy prevention, including menstrual suppression and gender affirmation. Options for menstrual suppression in transmasculine people include progestogen-only methods (pill, implant, injectable), the hormonal intrauterine system (e.g., levonorgestrel IUS) and GnRH analogues [4,5]. For those on a masculinising pathway, testosterone itself might supress bleeding. Combined hormonal contraceptives are generally avoided due to their oestrogen content. Testosterone therapy does not reliably prevent pregnancy and should not be relied upon as contraception [5]. Yet contraceptive research and clinical guidance have historically centred on cisgender women [6], leaving significant gaps in understanding how transmasculine people navigate contraceptive care [7]. This exclusionary practice leads to the varying needs of the individual not being fully met, and a better understanding is needed so that healthcare professionals can provide a holistic person-centred approach to contraceptive care.

Previous qualitative studies have explored influences on transmasculine people’s experiences of contraceptive methods and services in the USA [8,9,10,11]. A 2025 systematic review by Allen et al. highlighted that research into contraceptive barriers for transgender and gender-diverse people remains scarce, with most studies conducted in high-income countries, identifying key barriers including provider knowledge gaps, financial accessibility, systemic discrimination, and gender-biased healthcare practices [12]. Notably, no UK-specific qualitative research exists exploring transmasculine contraceptive experiences within the NHS, representing a significant gap given the distinct differences in access pathways, waiting times, and cultural context compared to the US settings that dominate the current literature. This study addresses that gap. Specifically, we sought to answer: (1) how do gender identity and dysphoria shape transmasculine people’s decision-making around contraceptive methods, and (2) what factors, within and beyond NHS healthcare services, influence transmasculine people’s experiences of accessing and using contraceptive methods in the UK?

## 2. Materials and Methods

We conducted individual interviews and a focus group with qualitative analysis. Interviews were chosen as they offer a meaningful insight into participants’ lived experiences and perspectives in a private setting [13]. A focus group was carried out as a method of exploring mutual experiences and feelings surrounding a topic [13], whilst also highlighting differing views between members of the studied group. A semi-structured topic guide was developed from existing literature and a prior qualitative evidence synthesis, with open-ended questions designed to encourage flexibility and depth. The topic guide was piloted with one participant prior to data collection and minor amendments were made to improve clarity. JM had prior experience conducting qualitative interviews through undergraduate research training and had completed a qualitative research methods module as part of her medical degree. RLM is experienced in qualitative research with under-represented communities. Interviews and a focus group were combined to capture complementary forms of insight. One-to-one interviews allowed participants to discuss sensitive and individual experiences, including disclosures they may not have felt comfortable sharing in a group setting, whilst the focus group was used to explore shared and divergent perspectives arising from peer discussion and interaction. The two data types were analysed within a single thematic framework, with focus-group data additionally examined for moments of interaction and disagreement between participants, which were treated as data rather than folded into individual quotations. The target sample size was not fixed in advance; recruitment continued for the planned study period, consistent with reflexive TA guidance that prioritises depth and richness of engagement with a small purposive sample over a pre-specified case count. The research team’s positionality was treated as an analytic resource throughout, rather than only a limitation to be noted afterwards. JM, RLM, and SB are white and cisgender, and none of the research team identified as trans or non-binary; JM, who conducted all interviews and the focus group, is also a medical student, which participants were aware of. These positions shaped both the interviewer-participant relationship and the interpretative lens brought to the data. Reflexive journaling and team discussion were used specifically to surface and interrogate assumptions arising from the team’s cisgender perspective, and to consider how a shared clinical/medical-student identity may have influenced what participants chose to disclose.

Ethical approval was granted by the University Research Ethics Committee (Application 063456). Inclusion criteria required participants to be aged 18 or over, able to speak and understand English, on the transmasculine spectrum, currently or previously using contraceptive methods, and to have accessed contraceptive care in the UK. This criterion was necessary to ensure participants could speak directly to their experiences of contraceptive services. However, it also means the sample cannot speak to the experiences of transmasculine people who have avoided contraceptive care entirely. For example, because of dysphoria, anticipated discrimination, or mistrust of services; given how central healthcare avoidance is to our findings, this is an important boundary of the sample that should be considered when interpreting the results. Given the sensitive nature of the topic, a distress and safeguarding procedure was agreed prior to data collection. JM, who conducted all interviews and the focus group, received a briefing from RLM on recognising and responding to disclosures of suicidality, self-harm, or disordered eating, and had access to supervision throughout the data collection period via scheduled check-ins. Participants were reminded at the start of each session that they could take a break or withdraw at any point without giving a reason. The protocol distinguished disclosures suggesting an acute or immediate risk of harm from accounts of past or non-acute distress; had an acute disclosure occurred, JM would have paused the interview, contacted RLM, and signposted the participant to emergency services. No disclosures met this threshold during data collection. All participants were offered signposting information at the end of the session through their GP regardless of whether distress had occurred. As a subset of participants were recruited via a youth support group, a pathway was agreed in advance for liaising with the organisation’s own safeguarding lead, with participant consent, should a serious concern arise.

Participants were recruited through purposive and snowball sampling over three months via local LGBTQ+ groups, University of Sheffield societies, and social media platforms such as LinkedIn. No relationship was established with participants prior to the study. Participants were informed that the research was being conducted by a medical student at the University of Sheffield as part of an intercalating research project, and that the study aimed to explore transmasculine people’s experiences of contraceptive methods and services in the UK.

Participants were located predominantly in South Yorkshire. JM conducted six interviews and one focus group (n = 4) at a local LGBTQ+ youth support group in South Yorkshire. We restricted recruitment to trusted community networks to reduce the risk of imposter participants and maintain data integrity. We recognise, however, that this approach is also likely to have contributed to the homogeneity of our sample (all white British and drawn from a single region), and we return to this as a limitation in Section 4.

We recruited ten participants: six in individual interviews and four in the focus group. The focus group lasted 45 min; interviews lasted between 23 and 57 min. One-to-one interviews were conducted remotely via video call to maximise accessibility and participant comfort. No non-participants were present during the one-to-one interviews. The focus group was conducted within a community LGBTQ+ youth group setting; no non-participants were present beyond the facilitator and participants. Reflective field notes were made by JM following each interview and focus group, capturing initial impressions, emerging ideas, and reflexive thoughts to support rigour throughout the analytical process.

Three individuals who expressed initial interest did not respond to follow-up contact and were therefore not recruited. No participants withdrew once the study had commenced. Repeat interviews were not conducted; each participant took part in a single interview or focus group only.

All participants were white British and aged between 18 and 27. Gender identities included transgender men, non-binary, agender, genderqueer, and gender non-conforming individuals (see Table 1).

Audio recordings were transcribed verbatim and anonymised. Transcripts were not returned to participants due to the risk of re-traumatisation and the preference expressed by participants during consent discussions for a single point of contact. Findings were not formally member-checked; however, reflexive journaling and regular team discussions were used throughout to interrogate interpretations and minimise researcher bias.

Data was managed and coded using NVivo^TM^ (version 15). Coding followed Braun and Clarke’s [14] reflexive thematic analysis approach, as further developed in their subsequent methodological writing [15,16]. Codes were generated inductively from participants’ language and experiences developed into themes through iterative, interpretative engagement with the dataset by JM, RLM, and SB. Given the small purposive sample and the exploratory nature of this study, data saturation in the traditional sense was not the aim. Rather, consistent with reflexive TA, the goal was theoretical sufficiency and depth of interpretation, in keeping with recommended sample-size rationales for this approach [16]. Coding and theme development were not treated as a means of establishing inter-rater reliability; instead, discussion between the three researchers was used as an analytic resource, with differing interpretative perspectives helping to enrich and test the developing themes (Table 2). Reflexive journaling and regular team discussions supported rigour throughout.

## 3. Results

**Figure 1:**
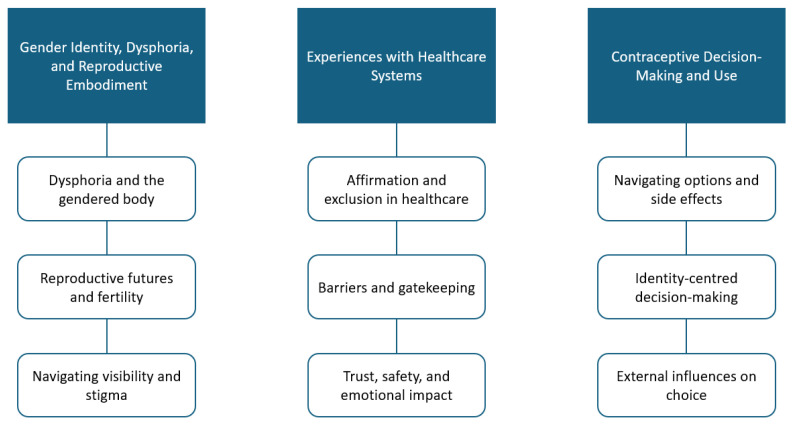
Hierarchy of themes.

Three overarching themes emerged, consistent with existing qualitative literature in this area and presented in Figure 1: (1) Gender Identity, Dysphoria, and Reproductive Embodiment; (2) Experiences of Healthcare Systems; and (3) Contraceptive Decision-Making and Use.

Theme 1: Gender Identity, Dysphoria, and Reproductive Embodiment

Participants’ experiences of contraceptive methods were shaped by their gender identity and embodied relationship with their reproductive organs. For many, menstruation, fertility, and contraceptive methods triggered or intensified gender dysphoria, reflecting a disconnect between identity and the socially constructed expectations surrounding people assigned female at birth. This theme encompassed two related subthemes shown in Figure 1: reproductive futures and fertility, and the broader embodied experience of dysphoria in relation to menstruation and pregnancy. These experiences were widely, though not universally shared across participants.

Several participants described menstruation as a painful reminder of their sex assigned at birth, intensifying feelings of dysphoria and disconnection from their bodies. For some, this discomfort reached crisis level. The two quotes below disclose experiences of acute distress; to further reduce the risk of re-identification within a small, close-knit participant group, these have been anonymised without pseudonym attribution, unlike other quotations in this paper.

*“I would start feeling very suicidal whenever I was on my period.”—Anonymised quote*

*“There was a time where I thought I’d been inconsistent enough with [my pills] that a period had started, and I fully went non-verbal for a while and I haven’t done that in over a year.”—Anonymised quote*

Others expressed discomfort that was deeply rooted in gendered expectations:

*“I think I don’t see myself as a woman in that way. To have periods feels too much like that.”—Morgan, age 23*

Some participants went to considerable lengths to prevent menstruation altogether. One participant described how fears around bleeding influenced their eating behaviours, connecting contraceptive use to wider disordered patterns of self-management. For others, period suppression through contraception was experienced as affirming:

*“I think it is kind of gender-affirming in that I feel more comfortable in my body not having to experience that every month.”—Morgan, age 23*

Pregnancy was similarly described as fundamentally incompatible with many participants’ gender identity:

*“Since realising about my gender identity, there’s also been the fact that obviously pregnancy is very much associated with womanhood and so the idea of getting pregnant now and actually going through with a pregnancy, it just doesn’t seem like something that I could ever handle.”—Miles, age 27*

In this context, permanent solutions like hysterectomy were sometimes framed as the most affirming and desirable outcome, though clinicians were perceived as resistant:

*“Doctors are very resistant to permanently never having children in a way they are for almost nothing else.”—Aspen, age 18*

*“Think less of the future children that may not be, and more of the person in front of you.”—Aspen, age 18*

Not all participants wished to avoid pregnancy entirely. One participant expressed a strong desire to carry children in the future, yet encountered stigma from peers who questioned whether this desire was compatible with a transmasculine identity:

*“My own mate is gaslighting me a bit like ‘okay you must be a woman then because you want to have a kid and carry it.’“—Cameron, age 22*

Navigating visibility and stigma was a further dimension of this theme. Participants described how stigma (experienced directly or anticipated) shaped their comfort and confidence in navigating reproductive services:

*“There’s a lot of stigma about it, like trans people coming in for contraception and stuff like that… if a trans guy comes in and is like ‘I want the pill’ or if worse comes to the worst, needs the morning after pill, there’s so much stigma, like you’re a guy—why do you need it?”—Kai, age 25*

The emotional labour involved in deciding whether to disclose gender identity in healthcare settings was significant; for some, withholding information felt like a protective strategy:

*“I think it’s not something you always feel comfortable with telling a doctor that you are non-binary because then there’s more questions behind that and kind of is easier to just pretend.”—Morgan, age 23*

Theme 2: Experiences of Healthcare Systems

Participants’ narratives highlight the complexity of navigating contraceptive care within healthcare systems that often fail to recognise or affirm their gender identities. Whilst some described instances of respectful and affirming care, many reported significant barriers, including healthcare professionals’ lack of knowledge, systemic gatekeeping, and discriminatory practices. Systemic gatekeeping referred to institutional policies and clinical practices that restricted access to care. For example, delays due to complex service pathways, an overreliance on binary gender categories in medical records and forms, and healthcare professionals acting as gatekeepers to certain methods. Within this theme, participants’ accounts clustered around trust, safety, and emotional impact (Figure 1): whether services felt safe enough to disclose gender identity in, and the emotional consequences—relief, resentment, or guardedness—of how that disclosure was received.

Even when contraceptive methods were chosen for gender-affirming reasons, participants were often left to navigate their implications without sufficient support or guidance. Experiences of misgendering and deadnaming disrupted what should have been routine appointments:

*“I do get misgendered. I do get misgendered though, by doctors. And deadnamed, even though it’s in the ‘nickname’ [section on the system].”—Alex, age 22*

The gendered nature of contraceptive services and information was a recurrent source of alienation:

*“I think a lot of it is very gendered, and people might forget that the anatomy might not match up.”—Aspen, age 18*

*“Everything’s just very gendered and… the leaflets all just use she/her pronouns, and I don’t like it.”—Jack, age 20*

*“And you look on the NHS website and it’s still using women and it’s uncomfortable to read the information that’s in the medication I’m taking.”—Jack, age 20*

One participant ultimately opted to access care privately after feeling invisible in NHS services:

*“They have ignored [my identity], completely ignored it, so I went private.”—Jasper, age 20*

These experiences were not isolated; participants highlighted structural issues across the healthcare system where their identities were disregarded or erased, with one noting:

*“I mean, the whole NHS system isn’t built with trans people in mind”—Jack, age 20*

Despite these challenges, participants reported positive encounters, especially in university-based healthcare settings, where they felt more able to be open about their identity and were met with respectful and responsive care:

*“I went through the university healthcare service and they’re very nice. I’ve had nothing but good experiences going through them for these problems.”—Jack, age 20*

Whilst these affirming experiences were welcomed, they also highlighted disparities in care. Some participants felt they could endure the discomfort, but resented that doing so was necessary in the first place:

*“I know that I can put up with it, but I don’t feel like I should have to put up with it.”—Jack, age 20*

Participants offered clear suggestions for improvement, centred on inclusive language and individualised care:

*“I don’t think I would care what gender people are. I think it’s just case-by-case and you just go for what’s best for that specific person. You don’t really think about anything else.”—Morgan, age 23*

Theme 3: Contraceptive Decision-Making and Use

Participants’ experiences of choosing and using contraceptive methods were shaped by a complex interplay of physical needs, gender identity, and external influences. Whilst some sought contraceptives to prevent pregnancy, others aimed to stop menstruation or reduce gender dysphoria. Choosing a method often involved weighing side effects, tolerability, and alignment with their identity. External influences on choice (Figure 1)—gendered marketing, peer and online networks, and uncertainty about hormone interactions—ran throughout this theme, frequently substituting for guidance participants felt they could not get from healthcare professionals.

Hormonal methods containing oestrogen were often ruled out entirely, viewed as directly incompatible with identity, both symbolically and physiologically:

*“I definitely can’t take oestrogen.”—Jack, age 20*

*“I wouldn’t ever want to use anything with oestrogen in.”—Miles, age 27*

Even progesterone-based methods or those that were not visibly feminising still carried associations with femininity that participants found emotionally difficult to navigate. The gendered marketing and language surrounding contraception further contributed to feelings of exclusion:

*“I can only look at cis things and the only male ones for genetic males are condoms that I know of. So, I’m like it’s all labelled as cis women stuff.”—Cameron, age 22*

*“The pill and stuff like that, it is more gendered. So, I don’t know about you lot but when I were on it when I realised I was trans, I kind of felt guilty because I felt like I was taking away a service for women because it’s highly gendered.”—Avery, age 21*

Side effects were a significant concern and influenced decisions profoundly. Mood changes, weight gain, and physical symptoms such as prolonged bleeding were commonly described:

*“I was basically having to wear sanitary towels more often than not just because if I didn’t, my pants would get covered in blood… it’s a year later—why is this still a problem?”—Miles, age 27*

*“[The dream contraceptive is] something that just didn’t affect me hormonally. It’s not going to affect my mood. It’s not going to affect my medication. It’s not going to affect my testosterone or oestrogen levels.”—Jack, age 20*

Pelvic procedures caused particular anxiety for some:

*“I wouldn’t ever consider an IUD because I would be very uncomfortable with the process to have one of those put in. I think that’s just a complete no.”—Aspen, age 18*

Methods that allowed for discretion and minimal engagement with reproductive anatomy were often preferred. One participant described preferring the implant precisely because of its invisibility:

*“With the implant, you literally don’t know that it’s there. And I think it just makes me more comfortable personally that only the people I’m really close to actually know I have one.”—Morgan, age 23*

Many participants encountered limited or unclear guidance from healthcare professionals and instead turned to peers, online communities, or other personal networks for advice. These informal sources played a key role in shaping decisions, especially when trust in professionals was low. Participants also highlighted the gendered imbalance in contraceptive side effects and responsibility:

*“When we’re on about side effects, it’s like assigned female at birth their style contraceptive have got all the side effects and all this, whereas assigned male at birth it’s like here’s a condom and that’s it.”—Kai, age 25*

Uncertainty around how contraceptives might interact with testosterone therapy was a persistent background concern:

*“Is it affecting my testosterone? Because I feel like there hasn’t been really that much research into it… it’s always just a bit of a worry in the back of my head, but it’s weighing up different things and I’d rather not get pregnant.”—Jack, age 20*

Overall, these accounts reflect how navigating contraceptive options is not only a matter of managing medical side effects but of managing dysphoria, autonomy, and fairness. For many, the limited range of acceptable options left them settling for less-than-ideal compromises:

*“It doesn’t really feel like there’s many good options.”—Jack, age 20*

## 4. Discussion

To our knowledge, this is the first UK-based qualitative exploration of transmasculine and non-binary people’s contraceptive experiences, drawing on interviews and a focus group with ten participants. Three overarching themes were identified, illustrating how contraceptive methods are experienced as deeply embodied, emotionally significant, and structurally constrained. These findings align with and extend prior work from the USA and other high-income countries [12], whilst contributing new insights grounded in the specific cultural and healthcare realities of the UK.

Consistent with existing literature [6,17,18,19], misgendering, healthcare professionals’ incorrect assumptions, and structural exclusion emerged as pervasive barriers in reproductive healthcare [20]. This study extends those findings by demonstrating how contraceptive methods themselves carry gendered meanings that influence feelings of dysphoria or identity misalignment. These findings echo studies from the US where deterrents to care included misgendering, feminised healthcare environments and discrimination [8,9,10,11].

These barriers can be explained through the lens of cisnormativity and cisgenderism [3], which position cisgender embodiment and identity as the unquestioned default within healthcare systems, rendering transmasculine bodies and needs illegible within services designed around cisgender women. This helps explain why misgendering and gendered service design were experienced not as isolated lapses but as a structural feature of contraceptive care. Minority stress theory [21] offers a complementary explanation for the emotional weight participants attached to disclosure decisions: the anticipation of prejudice, and the vigilance this required, itself constituted a source of distress independent of any single negative encounter. Reproductive justice [17] extends this further by framing contraceptive access as a question of bodily autonomy and the right to make informed reproductive decisions free from coercion or structural constraint. Participants’ accounts of being steered towards or away from methods on the basis of assumed gender, rather than individual preference, sit squarely within this framing. Together, these frameworks suggest that the barriers identified in this study are the product of healthcare systems structurally organised around cisgender assumptions, rather than merely incidental gaps in provider knowledge.

Participants described pills, coils, and implants as gendered objects or routines that can reinforce dysphoria, illustrating that contraceptive decisions cannot be separated from gender affirmation and embodied experiences. Community knowledge-sharing emerged as a critical supplement, and at times substitute, to professional advice, consistent with broader LGBTQ+ health findings about the importance of trusted peer networks [22].

Whilst certain themes were widely shared, such as the emotional impact of misgendering or the influence of dysphoria on contraceptive preferences, participants’ views and experiences also diverged in important ways. Preferences around fertility, attitudes towards hormonal methods, and the level of comfort engaging with healthcare systems varied substantially. These differences highlight the importance of trans-informed care that avoids assumptions and treats each person as an individual with unique needs and goals.

Participants voiced uncertainty around how testosterone might impact reliability of contraception which was also described by Gomez et al. in the US [9]. Whilst evidence on contraceptive-testosterone interactions remains limited, clinicians can offer reassurance through individualised review, shared decision-making, and referral to specialists where appropriate (e.g., GIC or endocrinology) [4]. The paucity of research in this area itself represents a barrier to care [12].

Whilst the research in the US and UK bear many similarities as defined by Allen et al. [12], there are distinct challenges for transmasculine people navigating UK/NHS systems. Firstly, the NHS’s reliance on binary-gendered electronic health records means that even participants who had recorded a preferred name were still deadnamed in consultations. The record system itself resurfaces incongruent information at the point of care, rather than this being solely a matter of clinician behaviour. Secondly, the absence of trans-affirming reproductive health guidance was visible in the gendered framing of routine information sources: participants described NHS leaflets and website content as addressed exclusively to women. Finally, the layered, multi-stage nature of NHS referral pathways (GP to specialist SRH or gender services, often via a Gender Identity Clinic waiting list) compounds delay in ways distinct from the more fragmented, insurance-mediated US system. This project aims to extend Allen et al.’s critique from the level of individual clinical encounters to the level of NHS infrastructure, which includes the record systems, information materials, and referral architecture that structure care. This is consistent with Mawson et al.’s [23] broader account of primary-care SRH access barriers, and suggests that improving individual clinician competence, while necessary, will not on its own resolve barriers built into how NHS services are administered.

Important limitations must be acknowledged. All participants were white British, and the majority completed school level education, limiting the generalisability of findings. We used trusted networks and struggled with imposter participants when we opened invites more widely on social forums. Transmasculine people from racially minoritised, working-class, or disabled backgrounds may face additional or compounded challenges, including mistrust of healthcare systems, discrimination, and structural constraints, that were not fully reflected in this dataset. Additionally, as with many small-scale qualitative studies, the sample size was limited. However, the richness of the narratives obtained allowed for deep engagement with each participant’s story, revealing complex dynamics of identity, power, and choice that quantitative research often overlooks [13]. As noted in Methods, the analytic team (JM, RLM, SB) was entirely white and cisgender, with no trans or non-binary team members. Whilst we used reflexive practice to interrogate our assumptions throughout analysis, this composition may still have led to misunderstanding or misinterpretation of participants’ accounts, and is an important limitation of this study.

Implications for Practice and Policy

Findings converge on the need for gender-affirming, identity-informed contraceptive care, though given the size and homogeneity of the sample, these implications should be read as a starting point for practice and further research rather than as definitive guidance. Clinicians must understand that methods perceived as gendered or misaligned with masculinisation goals can be rejected, even if they are clinically effective. This requires moving beyond assumptions and creating spaces for patient-centred discussions that respect transmasculine people’s embodied realities and concerns. Policy-level reforms should address the structural barriers identified in this study, such as the binary gendering of electronic medical records and the lack of trans-affirming care guidelines, guided by community voices and lived experiences.

Clinicians must be aware that safe prescribing for transmasculine patients requires knowledge of both natal sex and current gender identity, including any hormone therapy. For example, drug interactions between oestrogen-containing contraceptives and testosterone can affect both efficacy and safety [4]. This underscores the significance of creating clinical environments where patients feel safe to disclose their full medical picture.

Medical and nursing curricula and professional training should incorporate trans-specific reproductive health content to better equip clinicians. NHS services, advocacy groups, and guideline developers may use these insights to inform inclusive care delivery and training, with the potential to reduce healthcare avoidance, improve contraceptive access and satisfaction, and increase trust in services among transmasculine users. Improving contraceptive access and autonomy for transmasculine people is not only a clinical and policy imperative, but a matter of reproductive justice.

We would hypothesise that addressing the structural barriers identified here (binary-gendered records, absent trans-affirming guidance, and gatekeeping referral pathways) could plausibly reduce healthcare avoidance and increase trust among transmasculine service users. However, this study was not designed to test that causal pathway, and these data cannot demonstrate that such changes would in fact produce those outcomes. Evaluating whether specific service reforms reduce avoidance or improve trust would require prospective, outcome-focused research following implementation.

## 5. Conclusions

This study demonstrates that contraceptive experiences of transmasculine people are inseparable from gender identity, dysphoria, and systemic barriers within healthcare. The findings highlight that contraceptive methods are not neutral objects; they are deeply intertwined with experiences of gender, identity, and power, and can both alleviate and exacerbate gendered distress depending on how they are accessed, discussed, and delivered. By centring the voices of transmasculine people in the UK, this research contributes important new evidence to an underexplored area of reproductive healthcare and underscores the need for non-assumptive, person-centred care that affirms the full diversity of transmasculine experiences. These conclusions are drawn from a small, demographically narrow sample (all participants were white British and drawn from one region), and should be understood as a foundation for further UK research across more diverse transmasculine communities, rather than as generalisable claims.

## Figures and Tables

**Table 1:** Participant characteristics and pseudonyms.

	Pseudonym	Age	Gender as Defined by Participants	Ethnicity	Currently Contraception?	Data Source
1	Cameron	22	Agender/trans masc	White British	Yes	Interview
2	Taylor	19	Non-binary	White British	Yes	Interview
3	Miles	27	Male	White British	Yes	Interview
4	Alex	22	Non-binary	White British	Yes	Focus group
5	Kai	25	Male	White British	No	Focus group
6	Jasper	20	Male	White British	No	Focus group
7	Avery	21	Genderqueer	White British	No	Focus group
8	Morgan	23	Non-binary	White British	Yes	Interview
9	Jack	20	Male	White British	Yes	Interview
10	Aspen	18	Genderqueer	White British	Yes	Interview

**Table 2:** Examples of themes and subtheme development.

Data Extract	Code	Subtheme	Theme
“I never want to have another pregnancy scare again. I want to find a contraceptive that is going to be as effective as possible.”	Aversion to pregnancy	Reproductive futures and fertility	1: Gender Identity, Dysphoria, and Reproductive Embodiment
“A lot of gender dysphoria is tied to the areas of the body related to baby-making. It might be harder for people to discuss those issues because they don’t want to think about it.”	Gender dysphoria	Dysphoria and the gendered body	1: Gender Identity, Dysphoria, and Reproductive Embodiment
“They must think that just because I’ve got a vagina, that means I’m a female.”	Gender stereotyping from Healthcare Professionals	Affirmation and exclusion in healthcare	2: Experiences with Healthcare Systems
“It’s hard enough for a trans guy or non-binary person to be like ‘actually, I do need this help’. Then to go access that service, and for them to be hit by brick wall, by brick wall, by brick wall, because of their identity—it’s not on.”	Barriers to accessing healthcare	Barriers and gatekeeping	2: Experiences with Healthcare Systems
“They’re just like, ‘What if you change your mind?’ It’s the exact same rhetoric people use with trans stuff. And I think that’s why it hurts—because I hear this all the time.”	Loss of autonomy	Trust, safety, and emotional impact	2: Experiences with Healthcare Systems
“It is kind of gender-affirming in that I feel more comfortable in my body not having to experience that every month.”	Contraceptive being gender-affirming	Identity-centred decision-making	3: Contraceptive Decision-Making and Use
“I’m part of forums so trans guys teaching other trans guys otherwise you will not get that education because there’s nowhere really to find it.”	Peer influence	External influences on choice	3: Contraceptive Decision-Making and Use

## Data Availability

The interview and focus-group transcripts generated during this study are not publicly available. Given the sensitive, identifiable nature of the data—including disclosures relating to mental health, drawn from a small and close-knit participant community—and in line with the terms of participant consent, the underlying data cannot be shared in order to protect participant confidentiality.
